# Effect of overwintering on survival and vector competence of the West Nile virus vector *Culex pipiens*

**DOI:** 10.1186/s13071-019-3400-4

**Published:** 2019-03-27

**Authors:** Constantianus J. M. Koenraadt, Tim W. R. Möhlmann, Niels O. Verhulst, Jeroen Spitzen, Chantal B. F. Vogels

**Affiliations:** 1 Laboratory of Entomology, Wageningen University & Research, PO Box 16, 6700 AA Wageningen, The Netherlands; 2 National Centre for Vector Entomology, Institute of Parasitology, Vetsuisse Faculty, University of Zürich, Winterthurerstrasse 266A, 8057 Zurich, Switzerland; 3 Department of Epidemiology of Microbial Diseases, Yale School of Public Health, 60 College Street, P.O. Box 208034, New Haven, CT 06510 USA

**Keywords:** Overwintering, Diapause, Survival, Mosquito, Vector competence, West Nile virus, Longevity

## Abstract

**Background:**

West Nile virus (WNV) is a mosquito-borne virus that is mainly transmitted among birds by *Culex pipiens* mosquitoes. The species *Cx. pipiens* consists of two biotypes named *pipiens* and *molestus*, which together can form hybrids. One of the major distinctions between the biotypes is their overwintering behaviour. Adults of biotype *pipiens* diapause during winter, whereas biotype *molestus* remains actively blood-feeding. Diapausing may affect survival and vector competence of biotype *pipiens*. The aims of this study were therefore to identify the biotype composition of diapausing *Cx. pipiens* mosquitoes, to quantify survival throughout the autumn and winter months, and to determine effects of overwintering on vector competence of emerging *Cx. pipiens* mosquitoes for WNV.

**Methods:**

Diapausing mosquitoes were collected at two typical overwintering locations in the Netherlands. A selection of *Cx. pipiens* mosquitoes was identified to biotype using real-time PCR. Survival of diapausing *Cx. pipiens* mosquitoes during autumn and winter was monitored by placing cages with either field-collected or laboratory-reared females in houses and sheds. Vector competence of field-collected (diapausing) and laboratory-reared (non-diapausing) *Cx. pipiens* mosquitoes was determined to gain insight in the effect of overwintering on WNV transmission.

**Results:**

The majority (92%) of diapausing *Cx. pipiens* females were identified as biotype *pipiens*. More than 70% of diapausing *Cx. pipiens* mosquitoes was able to survive for more than four months in sheds, whereas diapausing in houses resulted in 100% mortality in that same period. In contrast, non-diapausing *Cx. pipiens* biotype *pipiens* mosquitoes reared in the laboratory died within a week in both houses and sheds. Vector competence of *Cx. pipiens* mosquitoes that had diapaused during the autumn and winter months was comparable to non-diapausing laboratory-reared mosquitoes.

**Conclusions:**

This study confirms that the majority of *Cx. pipiens* mosquitoes in their typical overwintering site belongs to the *pipiens* biotype. It shows that more than two-third of diapausing *Cx. pipiens* mosquitoes is able to survive winter under sheltered winter conditions. Finally, vector competence for WNV of mosquitoes that emerge from overwintering sites is not affected by their relatively old age.

## Background

Although various species of mosquitoes have been implicated as potential vector of West Nile virus (WNV), the species *Culex pipiens* (Linnaeus, 1758) is considered the most important in Europe and elsewhere [1]. This is related to its ubiquitous presence, its feeding on birds as reservoirs of the virus, its high local abundance and its close association with the domestic environment. Moreover, various laboratory studies with *Cx. pipiens* lines from different origins have demonstrated that the species is a highly competent vector of the virus [2–4]. During the outbreak of WNV in the United States, *Cx. pipiens* was estimated to contribute to approximately 80% of the transmission of WNV to humans. Other *Culex* and *Aedes* species made up the remaining 20% [5].

*Culex pipiens* actually comprises a group of species and there is still much debate about the taxonomic status of the various members of this group [6]. The two biotypes named biotype *pipiens* and biotype *molestus*, are recognized as behaviourally and physiologically distinct entities in Europe [7]. Whereas the *molestus* biotype prefers below-ground breeding habitats, *pipiens* is generally more abundant in above-ground habitats [7, 8]. However, recent evidence indicates a high degree of above-ground breeding of the *molestus* biotype even at more northern latitudes in Europe [9, 10]. In addition, autogeny is characteristic for *molestus* and such behaviour is not observed in the *pipiens* biotype. Mating in confined spaces occurs within *molestus*, while *pipiens* mates outdoors in swarms. Also, the *molestus* biotype is considered mammophilic, whereas *pipiens* is more ornitophilic. Both biotypes can hybridize and hybridization rates can reach up to 18% [9, 11]. Hybrids are also thought to be more opportunistic in their feeding behaviour and therefore considered important bridge vectors for transmission of WNV from the bird population (enzootic cycle) to humans and animals (epidemic/epizootic transmission) [12, 13].

Another major distinction between the two biotypes is the fact that females of biotype *pipiens* can enter a pre-programmed period of arrested development, termed diapause, which is characterized by the arrest of ovariole maturation and absence of blood-feeding [14]. After mating in autumn, males of *Cx. pipiens* die [15]. On the contrary, females of biotype *molestus* remain active and may even cause nuisance by their continued blood-feeding [9]. For biotype *pipiens*, diapause offers a mechanism to bridge unfavourable conditions during winter. Similar to other *Culex* species, diapause occurs in the adult stage and the hormonal pathways that underlie the physiological changes, such as building up fat reserves, are triggered by lowered temperature and reduced day length [14, 16]. In spring, with increasing temperatures and day length, the reverse occurs, and the species can emerge *en masse* from its overwintering sites [15, 17]. Feeding behaviour and survival after emergence remain elusive, but nearly 20% of *Cx. pipiens* biotype *pipiens* that had terminated diapause was attracted to a mammal (rat) in a laboratory setting, while the remaining 80% was attracted to a bird [18]. In the field, proportions of blood-fed *Culex* females increased with diapause termination after accruing a certain number of degree-days after winter solstice [19].

In light of repeated epidemics of WNV, understanding the fitness consequences of overwintering behaviour is critical [19, 20]. Prior to the onset of winter, mosquito vectors may have become infected via vertical (transgenerational) transmission [21, 22]. In case of WNV, infected vectors are considered to be infected with the virus for life, and those individuals that thus survive the winter may initiate virus circulation in spring of the next year [23]. However, the vast majority of emerging mosquitoes in spring will be uninfected, and their physiological state at that point may either result in enhanced vector competence because of a weakened immune system [24], or in reduced vector competence, because selection upon diapausing mosquitoes has resulted in strong survivors in comparison with the population at the start of the winter season. Also their age may impact vector competence as mosquitoes are relatively old at the end of winter and may not survive the long extrinsic incubation period as a result of cool spring temperatures [25]. In the present study, we set out to investigate these hypotheses by investigating (i) the biotype composition of *Cx. pipiens* populations in their natural overwintering site; (ii) the effect of location and local climate on the survival of diapausing *Cx. pipiens* populations; and (iii) the impact of diapausing of *Cx. pipiens* on their vector competence for WNV.

## Methods

### Mosquito collections

**Fig. 1:**
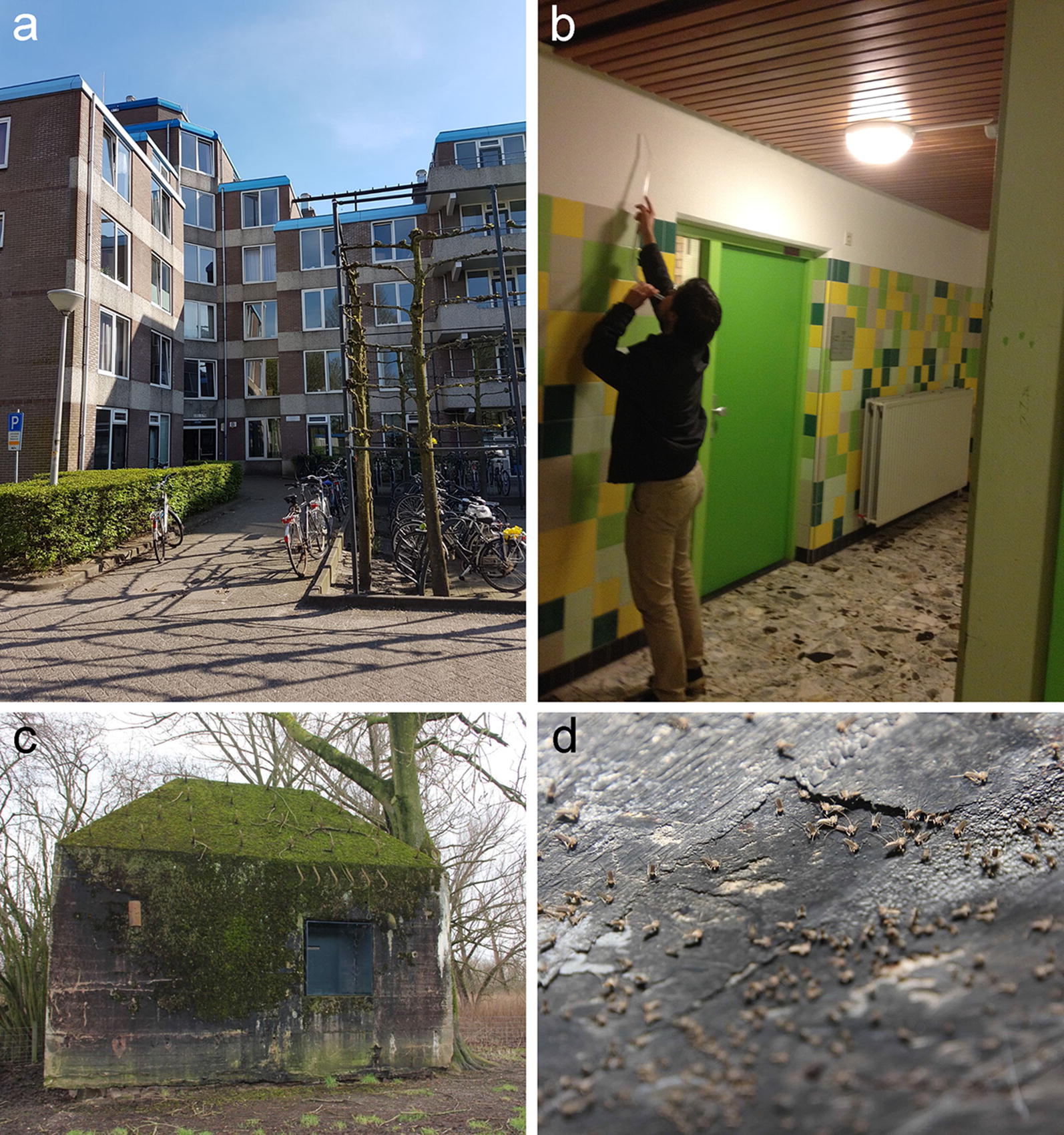
Sampling locations of overwintering mosquitoes. **a** Entrance of student apartment building in Wageningen, The Netherlands (Site 1). **b** Collecting mosquitoes in the entrance hall of Site 1. **c** One of the bunkers of the New Hollandic waterline near Asperen, The Netherlands (Site 2). **d** Overwintering mosquitoes inside one of the bunkers of the New Hollandic waterline. Source: **a**, Tim Möhlmann; **b**, Sander Koenraadt; **c** and **d**, Wiegert Steen

Adult mosquitoes were collected with an aspirator at two overwintering sites (Fig. 1). At Site 1, mosquitoes were collected on 21 October 2014 (daylength: 10 hours and 18 minutes) from the common entrances of a student apartment building in Wageningen, the Netherlands (Fig. 1a, b). At this site, numerous adult female mosquitoes with hypertrophied fat bodies were observed. These mosquitoes were most likely escaping the colder outdoor conditions of that day (T_min_ = 7.2 °C and T_max_ = 12.7 °C; [26]) and in search for a favourable overwintering site. At Site 2, mosquitoes were collected on 11 March 2015 from bunkers (coordinates: 51°52′31.7″N, 5°07′03.2″E and 51°52′17.2″N, 5°06′02.3″E) which are part of the new Hollandic waterline, near Asperen, the Netherlands (Fig. 1c, d). Mosquitoes use these sheltered bunkers as overwintering site. All mosquitoes were brought back to the Laboratory of Entomology of Wageningen University & Research for identification. Mosquitoes collected at Sites 1 and 2 were identified to species based on morphology using the key of Becker et al. [27]. *Culex pipiens* mosquitoes collected at Site 1 were used in a survival experiment in which longevity was monitored during the following autumn and winter months (late October-early March). A subset of 90 *Cx. pipiens* mosquitoes collected at Site 2 were further identified to biotype using a previously described real-time PCR method [9]. Vector competence was determined for mosquitoes that were alive after completion of the survival experiment and for a sub-set of 69 female *Cx. pipiens* mosquitoes collected at Site 2.

Laboratory-reared mosquitoes that were used in experiments were obtained from a colony of *Culex pipiens pipiens* that was established during the summer of 2014 as described previously [28]. This colony was maintained in the laboratory under summer conditions at 23 °C with 16:8 h light:dark photoperiod, and 60% relative humidity.

### Survival

Effects of overwintering environment and climate on survival were investigated for diapausing *Cx. pipiens* females collected in the field from overwintering Site 1 and for non-diapausing *Cx. p. pipiens* females reared in the laboratory. Field-collected and laboratory-reared mosquitoes were divided over six and four groups, respectively, of 30 ± 1 individuals in Bugdorm-1 insect rearing cages (30 × 30 × 30 cm; Bugdorm, Taiwan). Cotton wool moistened with water was added to each of the cages, and was moistened regularly to prevent dehydration. Cages with either field-collected or laboratory-reared mosquitoes were placed in two types of environment in three different locations. The first environment consisted of the inside of sheds, located up to 10 m away from human-inhabited and heated houses. These environments were exposed to ambient winter temperatures. The second environment consisted of unheated rooms in heated houses, such as attics, with a higher and more stable temperature than the sheds. Cages were placed on a table or shelf at a height of 1 to 2 m. The experiment was replicated in three houses and three sheds, with exception of the laboratory-reared mosquitoes which were only placed at two houses and two sheds. Minimum and maximum temperature, and relative humidity were recorded daily with instruments (Oregon Scientific Thermo-Hygro, Brea, CA, USA) placed next to the cages for each of the six locations.

### Vector competence

To gain insight in the effect of diapause on vector competence for WNV, we exposed field-collected and laboratory-reared *Cx. pipiens* females to WNV. Infection and transmission rates were determined for *Cx. pipiens* females collected at Site 1 (*n* = 14) that survived for > 125 days inside the sheds of the survival experiment carried out during the autumn and winter of 2014–2015, for *Cx. pipiens* females collected at overwintering Site 2 (*n* = 69) on 11 March 2015, and for laboratory-reared *Cx. p. pipiens* females as a control (*n* = 68). After being brought back to the laboratory, field-collected mosquitoes were kept under regular rearing conditions (23 °C and 16:8 L:D) for 8 days (Site 2) or 10 days (Site 1) until they were transferred to the Biosafety Level 3 facility and exposed to an infectious blood meal.

A passage 2 stock of WNV lineage 2 originating from Greece 2010 was grown on C6/36 cells. The stock virus with a 50% tissue culture infective dose of 8 × 10^7^ TCID_50_/ml was mixed with chicken blood (1:3 dilution) to a final titer of 2.7 × 10^7^ TCID_50_/ml. The infectious blood meal was provided through Parafilm M membrane using the Hemotek PS5 feeding system, in a dark room at 24 °C and 70% relative humidity. After one hour, mosquitoes were immobilised with 100% CO_2_ and fully engorged female mosquitoes were selected and placed in holding buckets (diameter: 12.2 cm, height: 12.2 cm; Jokey, Wipperfürth, Germany). Mosquitoes were maintained on 6% glucose solution ad libitum. After 14 days of incubation at 23 °C, saliva was collected as described in earlier studies [4, 28]. Mosquito body and saliva samples were stored at -80 °C, until being tested for presence of WNV. Frozen mosquito bodies were homogenized in the Bullet blender Storm (Next Advance, Averill Park, NY, United States), and saliva samples were thawed before being inoculated on a monolayer of Vero E6 cells in a 96-well plate. After six days, wells were scored for virus induced cytopathic effects. Presence of WNV in the mosquito body indicates successful infection of the mosquito body, and presence in the saliva indicates the potential for virus transmission.

### Statistical analysis

Data on survival of field-collected and laboratory-reared mosquitoes were analysed with Kaplan-Meier survival analysis. Multiple pairwise comparisons between the four groups (2 mosquito groups (field-collected or laboratory-reared) × 2 locations (inside houses or sheds)) were corrected using the Bonferroni correction. Infection and transmission rates were calculated as the number of virus-positive mosquito body or saliva samples, respectively, divided by the total number of surviving, virus-exposed mosquitoes, and multiplied by 100. Chi-square tests were used to test for significant differences in infection and transmission rates between field-collected and laboratory-reared mosquitoes. All statistical analyses were carried out with the statistical software package R [29].

## Results

### Survival during winter

**Fig. 2:**
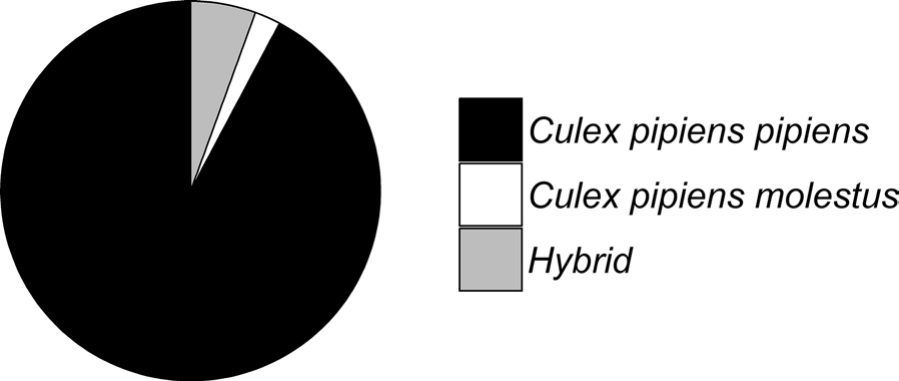
Biotype composition of 90 *Culex pipiens* mosquitoes collected in the bunkers of the Hollandic waterline in March 2015

Diapausing mosquitoes were collected from their natural overwintering site on 11 March 2015 and identified to species and biotype level. Out of the total number of 120 female mosquitoes, 77% was identified as *Cx. pipiens*, 7% as *Cx. territans*, 11% as *Culiseta annulata*, and 5% as *Anopheles maculipennis.* Of 90 *Cx. pipiens* females used for further analysis, 92.2% was identified as biotype *pipiens*, 2.2% as biotype *molestus*, and 5.6% as hybrids (Fig. 2). Thus, the majority of the *Cx. pipiens* mosquitoes that were collected in their natural overwintering habitat were indeed the *pipiens* biotype that is known to diapause during winter.

**Fig. 3:**
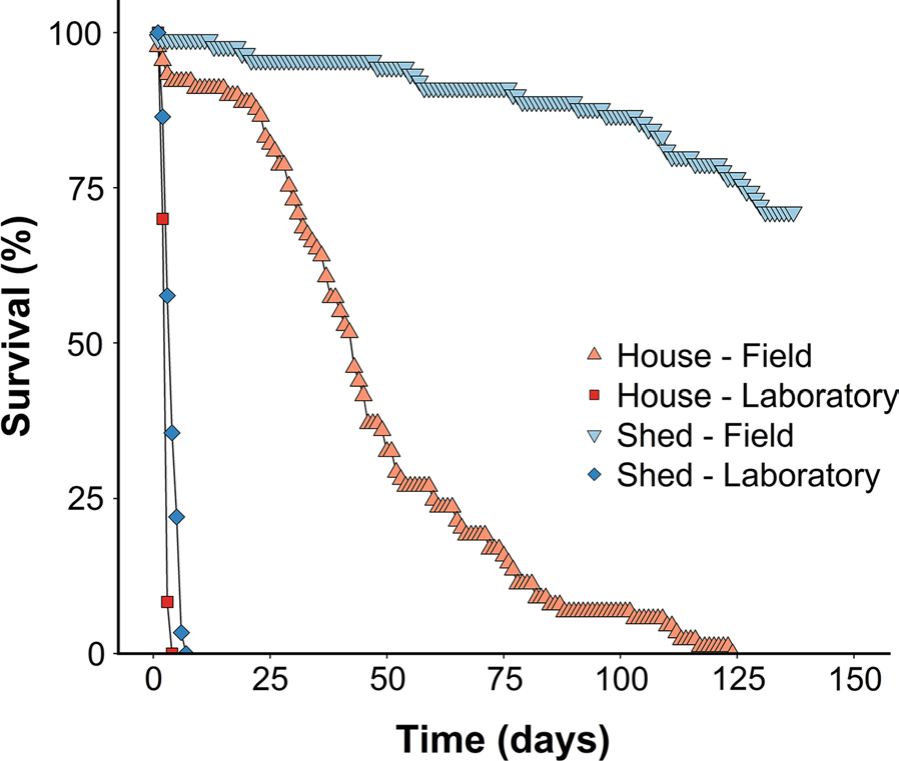
Percentage of surviving *Culex pipiens* mosquitoes over time. Survival of laboratory-reared and field-collected mosquitoes was monitored from 23 October 2014 to 9 March 2015 inside houses and sheds. The experiment was replicated in three different houses and sheds. Day 0 = 23 October 2014. Triangle, field-collected mosquitoes kept in houses (*n* = 89); square, laboratory-reared mosquitoes kept in houses (*n* = 60); inverted triangle, field-collected mosquitoes kept in sheds (*n* = 90); diamond, laboratory-reared mosquitoes kept in sheds (*n* = 59)

Survival of field-collected and laboratory-reared *Cx. pipiens* mosquitoes was monitored in houses and sheds from 23 October 2014 to 9 March 2015. Survival of all four groups was significantly different from each other (Kaplan-Meier survival analysis, all pairwise comparisons: *P* < 0.001). All laboratory-reared mosquitoes (non-diapausing) kept in houses died within four days, which was significantly faster than laboratory-reared mosquitoes that were kept in sheds which all died within seven days (Fig. 3). Thus, non-diapausing mosquitoes reared in the laboratory, were only able to survive for up to 6 days when deprived from nutritional resources.

**Fig. 4:**
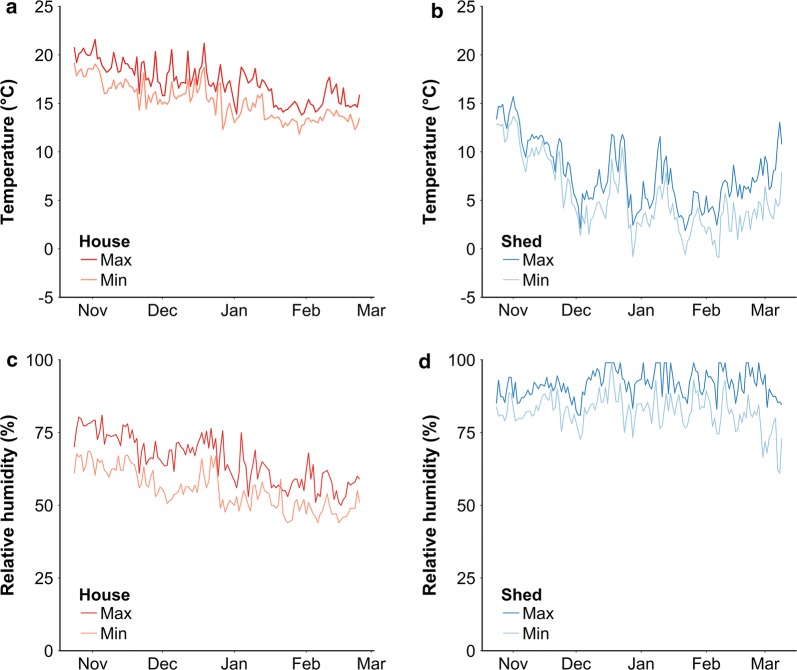
Climate data monitored during survival experiment from October 2014 to March 2015. Minimum and maximum temperature and relative humidity were recorded daily inside the houses and sheds of the survival experiment. Shown are minimum and maximum temperatures averaged for the three houses (**a**) and three sheds (**b**), as well as minimum and maximum relative humidity averaged for the three houses (**c**) and three sheds (**d**). Dark red, maximum values averaged for houses; light red, minimum values averaged for houses; dark blue, maximum values averaged for sheds; light blue, minimum values averaged for sheds

Diapausing *Cx. pipiens* mosquitoes that were collected when they were searching for a suitable overwintering site were able to survive for several months when only being provided moist cotton wool. Relatively higher temperatures and lower relative humidity, as experienced inside houses (Fig. 4a, c), negatively impacted survival of *Cx. pipiens* mosquitoes. Diapausing *Cx. pipiens* mosquitoes that were kept in houses died within 124 days, whereas only 30% of the mosquitoes that were kept in sheds died (Fig. 3). Thus, 70% of the diapausing mosquitoes was able to survive for more than 137 days in a typical overwintering environment.

Climate data were recorded daily for the duration of the survival experiment. Temperatures inside the three houses ranged between 8.8–23.7 °C (Fig. 4a), whereas temperatures inside sheds ranged between -2.7–17.0 °C (Fig. 4b). Relative humidity in houses ranged between 43–90% (Fig. 4c), whereas relative humidity in sheds ranged between 50–99% (Fig. 4d). Although temperatures in sheds fell below 0 °C on a few occasions, this did not result in increased mortality among mosquitoes exposed to these conditions.

### Vector competence for WNV

**Fig. 5:**
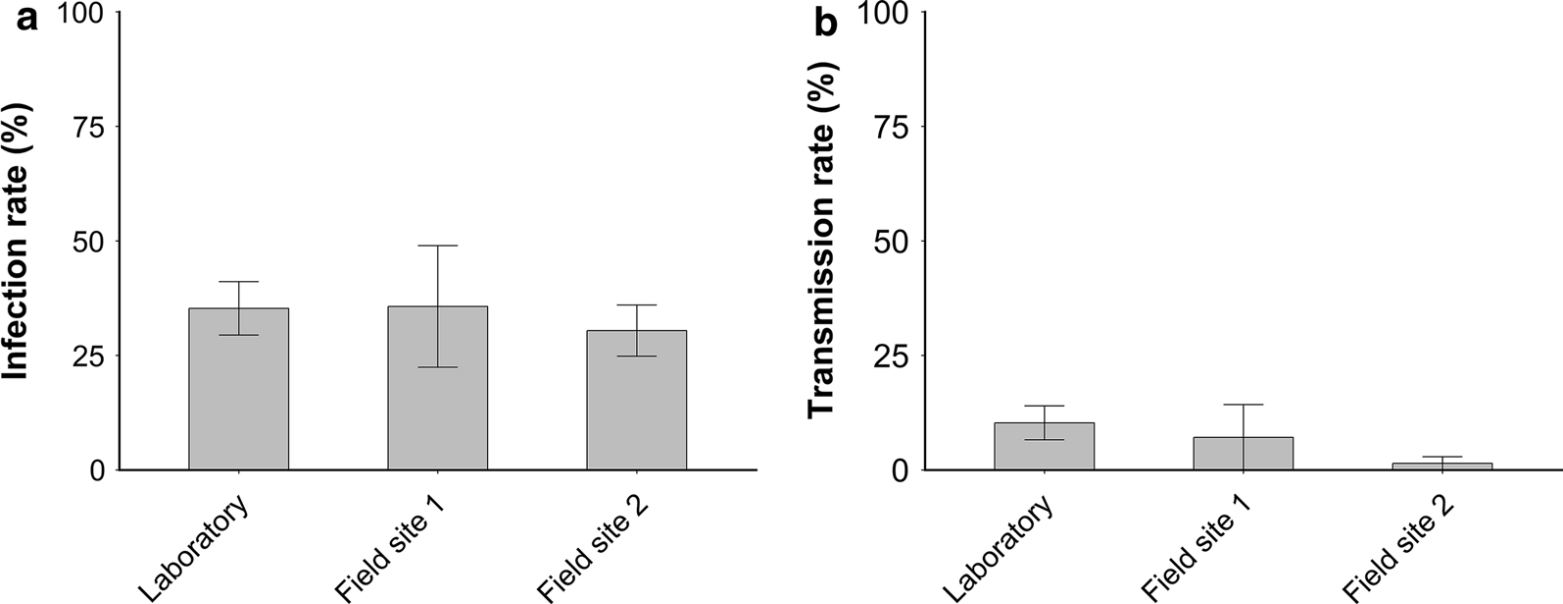
Vector competence of field-collected and laboratory-reared *Culex pipiens* mosquitoes for West Nile virus. **a** Mean infection rates of laboratory-reared and field-collected *Cx. pipiens* mosquitoes collected at overwintering Sites 1 and 2, orally exposed to WNV. **b** Mean transmission rates of laboratory-reared and field-collected *Culex pipiens* mosquitoes collected at overwintering Sites 1 and 2, orally exposed to WNV. Sample size for laboratory-reared (*n* = 68), overwintering Site 1 (*n* = 14; remaining mosquitoes survival experiment), and overwintering Site 2 (*n* = 69). Error bars indicate the standard error of the mean. Data were analysed with Chi-square tests. No significant differences were found between infection or transmission rates of the three groups

Vector competence of *Cx. pipiens* mosquitoes that had diapaused for more than four months was compared with laboratory-reared *Cx. p. pipiens* mosquitoes that were reared under summer conditions. No significant differences were found among infection rates of laboratory-reared mosquitoes (Laboratory: 35.3%, *n* = 68), surviving mosquitoes from the survival experiment (Field Site 1: 35.7%, *n* = 14), and diapausing mosquitoes collected from the field (Field Site 2: 30.4%, *n* = 69; *χ*^2^ = 0.41, *df* = 2, *P* = 0.81; Fig. 5a). Also, no significant differences were found among transmission rates of laboratory-reared mosquitoes (10.3%, *n *= 68), surviving mosquitoes from the survival experiment (7.1%, *n* = 14), and diapausing mosquitoes collected from the field (1.4%, *n* = 69; *χ*^2^ = 4.82, *df* = 2, *P* = 0.09 Fig. 5b). Thus, we found no evidence for altered vector competence of *Cx. pipiens* that diapaused throughout the autumn and winter months.

## Discussion

*Culex pipiens* biotype *pipiens* was the main biotype found in a typical overwintering site in the Netherlands. Approximately 70% of diapausing *Cx. pipiens* mosquitoes collected from their natural overwintering site and subsequently exposed to sheltered conditions could survive for over four months in sheds. Mosquitoes that were not prepared for overwintering died within one week when they had no access to a source of nutrition. No impact of prolonged survival during the winter months on vector competence of *Cx. pipiens* mosquitoes for WNV was found.

During winter *Cx. pipiens* biotype *pipiens* enters diapause to survive the cold months, and does no longer host seek [30]. While in diapause, responsiveness of the peripheral chemoreceptors to host-derived cues is reduced. This is exemplified by the fact that grooved peg sensilla located on the antennae of females are no longer sensitive to lactic acid [31]. Only when there is forced contact between mosquito and host, and the host-seeking step is thus skipped, a blood meal might be taken [30]. However, this blood is not used for the production of lipid reserves [32]. In contrast, biotype *molestus* remains actively blood feeding or persists as quiescent larvae [33, 34]. Typical overwintering habitats that biotype *pipiens* females select include sheltered places inside buildings, barns, or more natural settlements with a relatively mild and constant climate [7]. It should be noted that in our experiment mosquitoes were not able to select the most optimal micro-climate themselves as they were restricted to the cages that were placed in the different spaces. *Culex pipiens* mosquitoes sampled from bunkers mainly belonged to the *pipiens* biotype, whereas a few biotype *molestus* and hybrid mosquitoes were found as well. Presence of bats and rodents that seek shelter in these bunkers may constitute a source of blood which could provide nutrition for the *molestus* biotype. Overwintering bats were indeed observed during collections, although no blood fed mosquitoes were actually observed among the thousands of mosquitoes resting on the concrete walls of the bunker (Fig. 1d). Presence of hybrids could indicate their ability to go into diapause, although further investigation of their fat metabolism and ovarian maturation is required.

Our biotyping method employed an adapted diagnostic assay [9] that is based on a single locus (5′ flanking region of CQ11), and which assigns the specimens into three discrete groups (biotype *molestus*, biotype *pipiens* or hybrids thereof). In reality, the genetic structure of the *Culex pipiens* species is more complex, and other assays (such as the use of a microsatellite panel [35]) could shed more light on the genetic and associated phenotypic structure of the populations that we detected at the various sites. Although some of the biotypes may thus have been misclassified, the CQ11 assay is generally considered a reliable diagnostic method [36].

Several studies have investigated the physiology of mosquito diapause [14–16], and few of these attempted to quantify the proportion of wild *Cx. pipiens* mosquitoes that is able to survive a natural winter (reviewed in [15]). High mortality, up to 100%, was observed in *Cx. pipiens* populations from Russia, which was associated with average monthly temperatures of -3 °C. Similar high mortalities were observed in other areas of Russia, as well as in England, Germany and the USA [15]. Interestingly, experimental cold storage of mosquitoes that were not induced into diapause and that were placed at 6 °C led to 100% mortality within four months [37]. In our experiment, a similar average temperature (6.4–7 °C in the three different sites), but with *Cx. pipiens* mosquitoes naturally induced into diapause resulted in only 30% mortality. This is in line with earlier work that reported survival of 50% for six months in a diapause-induced *Cx. pipiens* colony exposed to simulated winter conditions in a laboratory [32]. Relatively colder and more humid conditions, as experienced in the sheds, resulted in the lowest mortality of diapausing mosquitoes. This is also in line with laboratory observations whereby the highest survival is seen at the lowest temperature for several *Culex* species, including *Cx. pipiens* [38]. Longitudinal studies on survival of *Cx. pipiens* biotypes and hybrids are needed to gain more detailed insights in dynamics of mosquito populations throughout the winter months across several years. This study provides important estimates for the proportion of *Cx. p. pipiens* that is capable of surviving a winter, and these estimates can be used for further refinement of risk models on viruses transmitted by *Cx. pipiens* mosquitoes.

No differences were found between infection or transmission rates of field-collected and laboratory-reared *Cx. pipiens* mosquitoes for WNV. Infection rates of 30–35% and transmission rates of 1–10% are comparable to rates reported for *Cx. pipiens* populations throughout Europe (reviewed in [1]). This suggests that *Cx. pipiens* mosquitoes that survived winter are not ‘weaker’ than their summer counterparts and thus more susceptible to WNV infection, nor are they ‘stronger’ and thus less susceptible to WNV infection as a result of a selection for those individuals that best survive the harsh winter conditions. It is known that insect immunity is variable throughout the year and in particular affected during overwintering [24], but in our study this did not affect WNV infection and transmission rates. The question remains to what extent (uninfected) mosquitoes that have overwintered will be exposed to WNV infected hosts in the field and thus potentially contribute to virus transmission. Although females readily blood-fed in our vector competence assay, we did not confirm whether those females emerging from diapause would actively seek hosts, which is an essential step prior to feeding. Although blood-fed females are frequently observed early in spring after diapause termination [19], it may well be that mosquitoes emerging from their winter hibernacula may not host seek, and lay eggs with their reserves only.

We did not investigate the effect of WNV infection on winter survival itself, and thus also not the likelihood of transmission being initiated very early in the season. In Europe, there is some evidence that WNV can be detected in overwintering mosquitoes [39]. WNV transmission cycles typically establish later in the season when high mosquito densities coincide with relatively high summer temperatures, although the transmission cycle also depends on host preference (bird *versus* mammal feeding) and the likelihood of host switching as determined by host availability [18, 40].

*Culex pipiens* mosquitoes that survived the duration of the survival experiment were at least five months old when being offered an infectious blood meal containing WNV. Unfortunately, low numbers of mosquitoes were left to determine their vector competence for WNV. Therefore, additional mosquitoes were collected by the end of winter at overwintering Site 2. The age of diapausing *Cx. pipiens* mosquitoes collected at overwintering Site 2 was therefore less certain, but it is reasonable to assume that these mosquitoes had sought shelter by fall 2014, similar to those mosquitoes collected in October 2014 at Site 1. Combined results on vector competence of both groups of diapausing *Cx. pipiens* mosquitoes indicates no impact of age on their ability to transmit WNV.

## Conclusions

This study confirms that the majority of *Cx. pipiens* mosquitoes collected from a typical overwintering site in The Netherlands belongs to the *pipiens* biotype. More than 70% of diapausing *Cx. pipiens* mosquitoes is able to survive winter under sheltered winter conditions, and vector competence of mosquitoes that emerge from overwintering sites is not affected by their relatively old age.

## Abbreviations

PCR: Polymerase Chain Reaction
TCID_50_: 50% Tissue Culture Infective Dose
WNV: West Nile virus

## References

[1] Vogels CBF, Göertz GP, Pijlman GP, Koenraadt CJM (2017). Vector competence of European mosquitoes for west Nile virus. Emerg Microbes Infect.

[2] Kilpatrick AM, Fonseca DM, Ebel GD, Reddy MR, Kramer LD (2010). Spatial and temporal variation in vector competence of *Culex pipiens* and *Cx. restuans* mosquitoes for West Nile virus. Am J Trop Med Hyg..

[3] Amraoui F, Krida G, Bouattour A, Rhim A, Daaboub J, Harrat Z (2012). *Culex pipiens*, an experimental efficient vector of West Nile and Rift Valley fever viruses in the Maghreb region. PLoS One..

[4] Vogels CBF, Göertz GP, Pijlman GP, Koenraadt CJM (2017). Vector competence of northern and southern European *Culex pipiens pipiens* mosquitoes for West Nile virus across a gradient of temperatures. Med Vet Entomol.

[5] Kilpatrick AM, Kramer LD, Campbell SR, Alleyne EO, Dobson AP, Daszak P (2005). West Nile virus risk assessment and the bridge vector paradigm. Emerg Infect Dis.

[6] Harbach RE (2012). *Culex pipiens*: species versus species complex - taxonomic history and perspective. J Am Mosq Control Assoc..

[7] Becker N, Jost A, Weitzel T (2012). The *Culex pipiens* complex in Europe. J Am Mosq Control Assoc..

[8] Byrne K, Nichols RA (1999). *Culex pipiens* in London Underground tunnels: differentiation between surface and subterranean populations. Heredity.

[9] Vogels CBF, Van De Peppel LJJ, van Vliet AJH, Westenberg M, Ibañez-Justicia A, Stroo A (2015). Winter activity and aboveground hybridization between the two biotypes of the West Nile virus vector *Culex pipiens*. Vector Borne Zoonotic Dis..

[10] Rudolf M, Czajka C, Börstler J, Melaun C, Jöst H, von Thien H (2013). First nationwide surveillance of *Culex pipiens* complex and *Culex torrentium* mosquitoes demonstrated the presence of *Culex pipiens* biotype *pipiens*/*molestus* hybrids in Germany. PLoS One..

[11] Vogels CBF, Möhlmann TWR, Melsen D, Favia G, Wennergren U, Koenraadt CJM (2016). Latitudinal diversity of *Culex pipiens* biotypes and hybrids in farm, peri-urban, and wetland habitats in Europe. PLoS One..

[12] Fritz ML, Walker ED, Miller JR, Severson DW, Dworkin I (2015). Divergent host preferences of above- and below-ground *Culex pipiens* mosquitoes and their hybrid offspring. Med Vet Entomol.

[13] Fonseca DM, Keyghobadi N, Malcolm CA, Mehmet C, Schaffner F, Mogi M (2004). Emerging vectors in the *Culex pipiens* complex. Science.

[14] Denlinger DL, Armbruster PA (2014). Mosquito diapause. Annu Rev Entomol.

[15] Vinogradova EB (2000). *Culex pipiens pipiens* mosquitoes: taxonomy, distribution, ecology, physiology, genetics, applied importance and control.

[16] Sim C, Denlinger DL (2008). Insulin signaling and FOXO regulate the overwintering diapause of the mosquito *Culex pipiens*. Proc Natl Acad Sci USA.

[17] Ciota AT, Drummond CL, Drobnack J, Ruby MA, Kramer LD, Ebel GD (2011). Emergence of *Culex pipiens* from overwintering hibernacula. J Am Mosq Control Assoc..

[18] Faraji A, Gaugler R (2015). Experimental host preference of diapause and non-diapause induced *Culex pipiens pipiens* (Diptera: Culicidae). Parasit Vectors..

[19] Nelms BM, Macedo PA, Kothera L, Savage HM, Reisen WK (2013). Overwintering biology of *Culex* (Diptera: Culicidae) mosquitoes in the Sacramento Valley of California. J Med Entomol.

[20] Nelms BM, Kothera L, Thiemann T, Macedo PA, Savage HM, Reisen WK (2013). Phenotypic variation among *Culex pipiens* complex (Diptera: Culicidae) populations from the Sacramento Valley, California: horizontal and vertical transmission of West Nile virus, diapause potential, autogeny, and host selection. Am J Trop Med Hyg.

[21] Farajollahi A, Crans WJ, Bryant P, Wolf B, Burkhalter KL, Godsey MS (2005). Detection of West Nile viral RNA from an overwintering pool of *Culex pipens pipiens* (Diptera: Culicidae) in New Jersey, 2003. J Med Entomol.

[22] Fechter-Leggett E, Nelms BM, Barker CM, Reisen WK (2012). West Nile virus cluster analysis and vertical transmission in *Culex pipiens* complex mosquitoes in Sacramento and Yolo Counties, California, 2011. J Vector Ecol..

[23] Nasci RS, Savage HM, White DJ, Miller JR, Cropp BC, Godsey MS (2001). West Nile virus in overwintering *Culex* mosquitoes, New York City, 2000. Emerg Infect Dis.

[24] Ferguson LV, Sinclair BJ (2017). Insect immunity varies idiosyncratically during overwintering. J Exp Zool Part A Ecol Genet Physiol..

[25] Reisen WK, Fang Y, Martinez VM (2006). Effects of temperature on the transmission of West Nile virus by *Culex tarsalis* (Diptera: Culicidae). J Med Entomol.

[26] Koninklijk Nederlands Meteorologisch Instituut. http://www.knmi.nl. Accessed 26 Apr 2018.

[27] Becker N (2010). Mosquitoes and their control.

[28] Vogels CBF, Fros JJ, Göertz GP, Pijlman GP, Koenraadt CJM (2016). Vector competence of northern European *Culex pipiens* biotypes and hybrids for West Nile virus is differentially affected by temperature. Parasit Vectors..

[29] R Development Core Team (2016). R: a language and environment for statistical computing.

[30] Mitchell CJ (1983). Differentiation of host-seeking behavior from blood-feeding behavior in overwintering *Culex pipiens* (Diptera: Culicidae) and observations on gonotrophic dissociation. J Med Entomol.

[31] Bowen MF, Davis EE, Haggart DA (1988). A behavioural and sensory analysis of host-seeking behaviour in the diapausing mosquito *Culex pipiens*. J Insect Physiol.

[32] Mitchell CJ, Briegel H (1989). Inability of diapausing *Culex pipiens* (Diptera: Culicidae) to use blood for producing lipid reserves for overwinter survival. J Med Entomol.

[33] Spielman A, Wong J (1973). Studies on autogeny in natural populations of *Culex pipien*s. 3. Midsummer preparation for hibernation in anautogenous populations. J Med Entomol..

[34] Spielman A (1971). Studies on autogeny in natural populations of *Culex pipiens.* II. Seasonal abundance of autogenous and anautogenous populations. J Med Entomol..

[35] Gomes B, Sousa CA, Novo MT, Freitas FB, Alves R, Côrte-Real AR (2009). Asymmetric introgression between sympatric *molestus* and *pipiens* forms of *Culex pipiens* (Diptera: Culicidae) in the Comporta region, Portugal. BMC Evol Biol.

[36] Di Luca M, Toma L, Boccolini D, Severini F, La Rosa G, Minelli G (2016). Ecological distribution and CQ11 genetic structure of *Culex pipiens* complex (Diptera: Culicidae) in Italy. PLoS One..

[37] Rinehart JP, Yocum GD, Leopold RA, Robich RM (2010). Cold storage of *Culex pipiens* in the absence of diapause. J Med Entomol.

[38] Ciota AT, Matacchiero AC, Kilpatrick AM, Kramer LD (2014). The effect of temperature on life history traits of *Culex* mosquitoes. J Med Entomol.

[39] Rudolf I, Betášová L, Blažejová H, Venclíková K, Straková P, Šebesta O (2017). West Nile virus in overwintering mosquitoes, central Europe. Parasit Vectors.

[40] Farajollahi A, Fonseca DM, Kramer LD, Kilpatrick AM (2011). “Bird biting” mosquitoes and human disease: a review of the role of *Culex pipiens* complex mosquitoes in epidemiology. Infect Genet Evol.

